# Supplementary material for: A Horizontally Transferred Autonomous Helitron Became a Full Polydnavirus Segment in Cotesia vestalis
Source: G3 (Bethesda). 2017 Oct 17;7(12):3925–35. doi: 10.1534/g3.117.300280 (PMC5714489; doi:10.1534/g3.117.300280)
Supplement: Supplementary file 1 [file 3925FigureS1.pdf]

Hel_c35

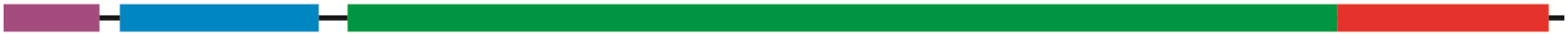

Name	Position	Similarity [%]
Helitron-N2_DBp	1-320	80.86
Helitron-N2_DAna	394-1058	94.61
Helitron-N2_DHMM	1159-4493	70.08
Helitron-N2_DBp	4494-5195	97.29

Figure S1. Hel_c35 shows similarity to several Helitrons from Repbase. Analysis performed with the CENSOR tool (Kohany *et al*. 2006) against the whole Repbase repeat library (Jurka *et al*. 2005). The CENSOR tool is available in http://www.girinst.org/censor/ (last accessed in February 2017).
