# Supplementary material for: A Horizontally Transferred Autonomous Helitron Became a Full Polydnavirus Segment in Cotesia vestalis
Source: G3 (Bethesda). 2017 Oct 17;7(12):3925–35. doi: 10.1534/g3.117.300280 (PMC5714489; doi:10.1534/g3.117.300280)
Supplement: Supplementary file 2 [file 3925FigureS2.pdf]

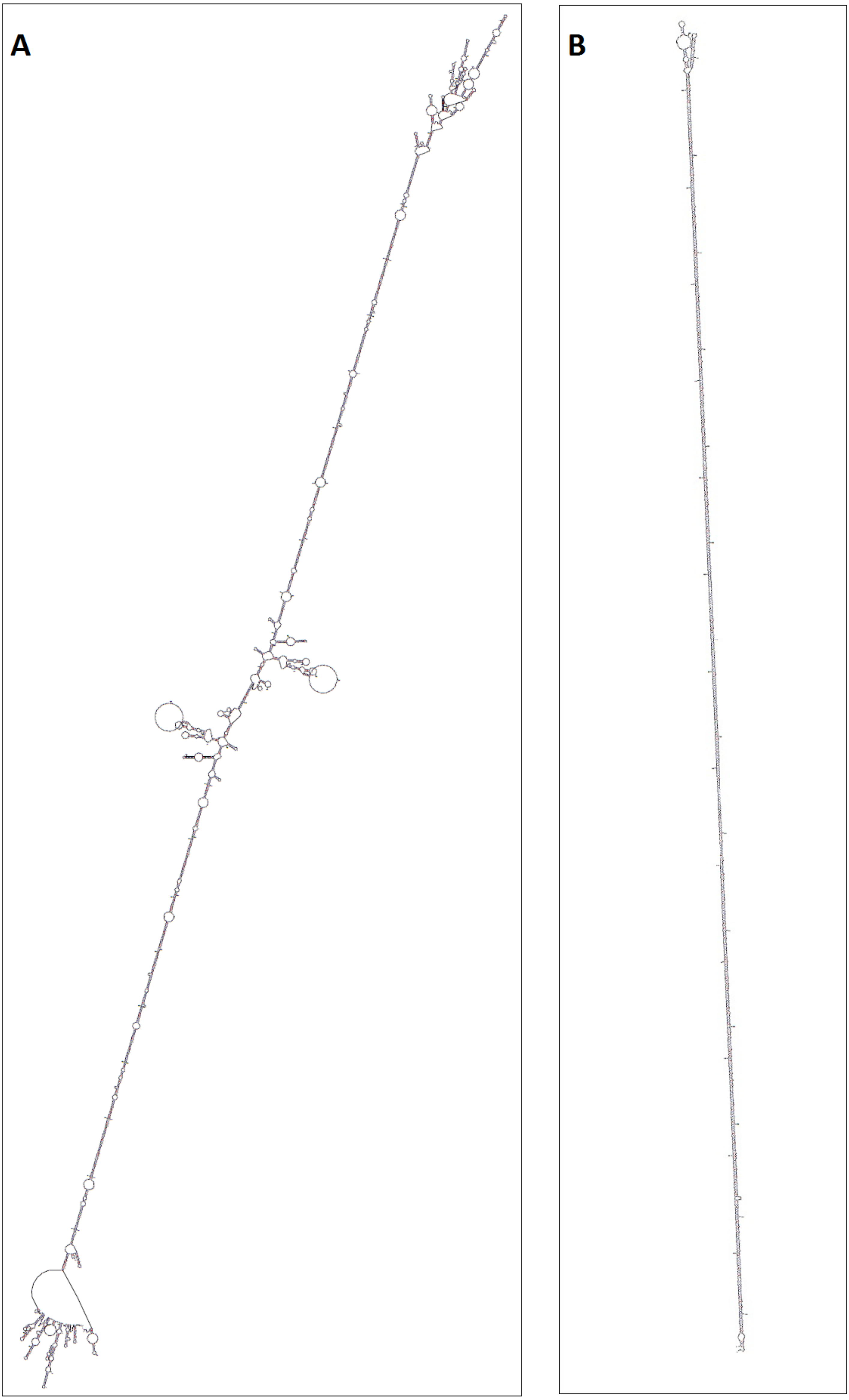

Figure S2. Predicted secondary structures upstream (A) and downstream (B) the Hel_c35 copy in the genome *Cotesia vestalis* genome, using the software mfold (Zuker 2003).
