# Supplementary material for: A Horizontally Transferred Autonomous Helitron Became a Full Polydnavirus Segment in Cotesia vestalis
Source: G3 (Bethesda). 2017 Oct 17;7(12):3925–35. doi: 10.1534/g3.117.300280 (PMC5714489; doi:10.1534/g3.117.300280)
Supplement: Supplementary file 3 [file 3925FigureS3.pdf]

```
CvBV_c35       CACGTTATTATACTTGGAATGCTTCATCGAAGAATTTTCAAAGACGGAAGCAAGGCGATGCGGTTC
Unigene42046   CACGTTATTATACTTGGAGTGCTTCATCGAAGAATTTTCAAAGACGGAAGCAAGGCGATGCGGTTC

cons           ****************** ***********************************************


CvBV_c35       CTGGGTATCCAGATGTGCGTTCTACTGATGCTCTTGGTCGTATGTATACAGTTCATCCAAAGAATA
Unigene42046   CTGGGTATCCAGATGTGCGTTCTACTGATGCTCTTGGTCGTATGTATACAGTTCATCCAAAGAATG

cons           ******************************************************************


CvBV_c35       ATGAATGTTTCTATTTGCGGTTGTTGCTGGTAAATGTGCGTGGCCAACGTCATTTGAGTCACTAC
Unigene42046   ATGAATGTTTCTATTTGCGGTTGTTGCTGGTAAATGTGCGTGGCCAACGTCATTTGAGACACTAC

cons           ********************************************************** ******


CvBV_c35       GAACTGTTAATGGTGTAATATTCCC
Unigene42046   GAACTGTTAATGGTGTAATATTCCC

cons           *************************
```

Figure S3. Sequence alignment of Hel_c35 and *Chilo suppressalis* transcripts. Sequences were aligned with default options in the M-coffee web server (Moretti *et al*. 2007; available in http://tcoffee.crg.cat/apps/tcoffee/do:mcoffee).
