# Supplementary material for: A Horizontally Transferred Autonomous Helitron Became a Full Polydnavirus Segment in Cotesia vestalis
Source: G3 (Bethesda). 2017 Oct 17;7(12):3925–35. doi: 10.1534/g3.117.300280 (PMC5714489; doi:10.1534/g3.117.300280)
Supplement: Supplementary file 4 [file 3925FigureS4.pdf]

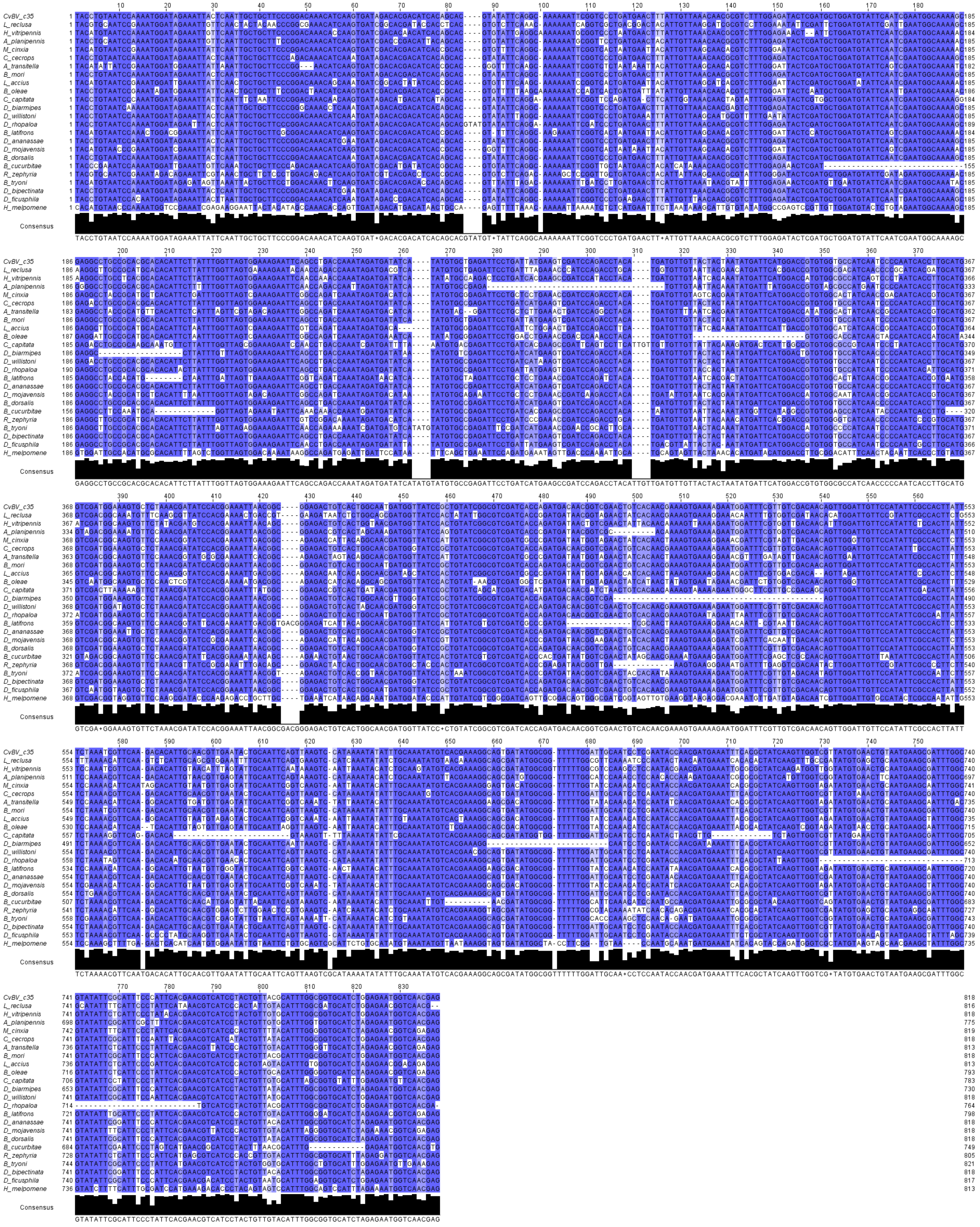

Figure S4. MUSCLE alignment of Hel_c35 Rep sequences from arthropod genomes. Alignment was conducted using MEGA7 (Kumar *et al*. 2016). Formatted on Jalview (Waterhouse *et al*. 2009).
