# Supplementary material for: A Horizontally Transferred Autonomous Helitron Became a Full Polydnavirus Segment in Cotesia vestalis
Source: G3 (Bethesda). 2017 Oct 17;7(12):3925–35. doi: 10.1534/g3.117.300280 (PMC5714489; doi:10.1534/g3.117.300280)
Supplement: Supplementary file 5 [file 3925TableS1.pdf]

Table S1. Bracovirus-related sequences flanking Hel_C35 insertion.

Conserved sequences	Distance from Hel_c35	BV with > 70% sequence identity hits
Upstream		
	16.9 kb	CcBV, CsBV
	9.5 kb	CvBV, CcBV, CsBV, GiBV, GfBV, MmBV
	1.18 kb	CcBV, CsBV
	0 kb	CsBV, CvBV*, CcBV*, GiBV*
Downstream		
	2.5 kb	CcBV, CsBV
	3.5 kb	CvBV, CcBV, CsBV,
	5.1 kb	CvBV, CcBV, CsBV

* Hits retrieved from adjacent sequences (see text)

Abbreviations: CcBV, *Cotesia congregata* bracovirus; CsBV, *Cotesia sesamiae* bracovirus; CvBV, *Cotesia vestalis* bracovirus; MmBV, *Microplitis mediator* bracovirus; GiBV, *Glyptapanteles indiensis* bracovirus; GfBV, *Glyptapanteles flavicoxis* bracovirus.
