# Supplementary material for: A Horizontally Transferred Autonomous Helitron Became a Full Polydnavirus Segment in Cotesia vestalis
Source: G3 (Bethesda). 2017 Oct 17;7(12):3925–35. doi: 10.1534/g3.117.300280 (PMC5714489; doi:10.1534/g3.117.300280)
Supplement: Supplementary file 6 [file 3925TableS2.pdf]

Table S2. Species used for alignment and phylogeny of Hel_c35 Rep sequences.

Taxon name	Abbreviation	Accession Number	Hit Position
**PDV**			
Cotesia vestalis bracovirus	*CvBV*	HQ009558.1	2814-4488
**Arachnida**			
Loxosceles reclusa	*L_reclusa*	JJRW010240300.1	357-1970
**Hemiptera**			
Homalodisca vitripennis	*H_vitripennis*	JJNS01106183.1	1533-3097
**Coleoptera**			
Agrilus planipennis	*A_planipennis*	JENH01003481.1	6673-8240
**Lepidoptera**			
Melitaea cinxia	*M_cinxia*	APLT01048631.1	1250-2526
Calycopis cecrops	*C_cecrops*	LUGF01005276.1	182703-184361
Amyelois transitella	*A_transitella*	LACK01030416.1	4096-5612
Bombyx mori	*B_mori*	BAAB01008891.1	669-2228
Lerema accius	*L_accius*	LGAG01000166.1	257040-258655
Heliconius melpomene	*H_melpomene*	CAEZ01011453.1	10643-12242
**Diptera**			
Bactrocera oleae	*B_oleae*	LGAM01001181.1	44683-45909
Ceratitis capitata	*C_capitata*	AOHK01022623.1	19163-20708
Drosophila biarmipes	*D_biarmipes*	AFFD02006852.1	1-1348
Drosophila willistoni	*D_willistoni*	AAQB01008178.1	12755-14338
Drosophila rhopaloa	*D_rhopaloa*	AFPP02022585.1	1867-2951
Bactrocera latifrons	*B_latifrons*	MIMC01001038.1	48994-50594
Drosophila ananassae	*D_ananassae*	AAPP01019267.1	78661-80027
Drosophila mojavensis	*D_mojavensis*	AAPU01002525.1	260-1881
Bactrocera dorsalis	*B_dorsalis*	JFBF01000201.1	312085-313742
Bactrocera cucurbitae	*B_cucurbitae*	JRNW01026949.1	687-2193
Rhagoletis zephyria	*R_zephyria*	LYWK01008048.1	17702-19306
Bactrocera tryoni	*B_tryoni*	JHQJ01005676.1	3693-5324
Drosophila bipectinata	*D_bipectinata*	AFFE02003548.1	4566-6223
Drosophila ficusphila	*D_ficusphila*	AFFG02008925.1	745-2390
