# Supplementary material for: A Horizontally Transferred Autonomous Helitron Became a Full Polydnavirus Segment in Cotesia vestalis
Source: G3 (Bethesda). 2017 Oct 17;7(12):3925–35. doi: 10.1534/g3.117.300280 (PMC5714489; doi:10.1534/g3.117.300280)
Supplement: Supplementary file 7 [file 3925FileS1.pdf]

File S1. Nucleotide sequence used as a query for searches on arthropod genomes. (A) Nucleotide sequence used as a query for Blastn searches on arthropod genomes. Sequence coding catalytic core from the Rep domain highlighted in green, and region coding flanking amino acid sequences in grey. The flanking N-terminal region in grey includes the rest of the Rep domain and the C-terminal region includes part of the sequence coding intermediate amino acids between Rep and Hel domains. (B) Complete Rep/Hel amino acid sequence with colors corresponding to the same marked regions from the nucleotide sequence. Pif1 helicase domain marked in yellow.

A

TATTGTTTGCCTTTAAAATTTGCCTTTGAAATGGTTAATTATCAAATTTTTAATTTCAGGTGAAGAAACTACAAAGAAAGTTAGCTC
AATGAATTATTACGCATATCGTTTGATGATTCGTCAAAATGCTGACAACTATTTGCTGCGGTTTCGTCGATTGTTTCAGCAGTATTG
CGTTGACATGTATGTAAAAATAGAAACGGAACGTTTAACATTCATTAGGTTGAACCAAGCCAAACTGCGTTCTGAGGAGTACATCCA
TTTACGTGATGCAGTTAATACTGAAGGAAATGCAGCTAATATTGGTCGATTAACTATTCTGCCGGCGACATACATTGGTAGTCCACG
TCATATGCATGAATATGCACAAGATGCAATGACATATGTTCGTCATTACGGTCGGCCCGATCTCTTTATTACTTTTACCTGTAATCC
AAAATGGATAGAAATTACTCAATTGCTGCTTCCCGGACAAACATCAAGTGATAGACACGACATCACAGCACGTATATTCAGGCAAAA
AATTCGGTCCCTGATGAACTTTATTGTTAAACAACGCGTCTTTGGAGATACTCGATGCTGGATGTATTCAATCGAATGGCAAAAGCG
AGGCCTGCCGCACGCACACATTCTTATTTGGTTAGTGGAAAGAATTCAGCCTGACCAAATAGATGATATCATATGTGCTGAGATTCC
TGATTATGAAGTCGATCCAGACCTACATGATGTTGTTACTACTAATATGATTCATGGACCGTGTGGTGCCATCAATCCCCAATCACC
TTGCATGGTCGATGGAAAGTGCTCTAAACGATATCCACGGAAATTAACGGCGGAGACTGTCACTGGCAATGATGGTTATCCGCTGTA
TCGGCGTCGATCACCAGATGACAACGGTCGAACTGTCACAACGAAAGTGAAAAGAATGGATTTCGTTGTCGACAACAGTTGGATTGT
TCCATATTCGCCACTTATTTCTAAATCGTTCAAGACACATTGCAACGTTGAATACTGCAATTCAGTTAAGTCCATAAAATATATTTG
CAAATATGTCACGAAAGGCAGTGATATGGCGGTTTTTGGATTGCAATCCTCGAATACCAACGATGAAATTTCACGCTATCAAGTTGG
TCGTTATGTGAACTGTAATGAAGCGATTTGGCGTATATTCGCATTTCCCATTCACGAACGTCATCCTACTGTTACGCATTTGGCGGT
GCATCTGGAGAATGGTCAACGAGTATATTTCACGGCTTCGAATGCTACGCAACGTGCTGAAACACCTCCAGCAACTACATTGACCAG
TTTTTTTGCAATCTGCCAAAGCGATCAGTTTGCACGAACTTTGCTTTACTCGGAGATGCCACGTTATTATACTTGGAATGCTTCATC
GAAGAATTTTCAAAGACGGAAGCAAGGCGATGCGGTTCCTGGGTATCCAGATGTGCGTTCTACTGATGCTCTTGGTCGTATGTATAC
AGTTCATCCAAAGAATAATGAATGTTTCTATTTGCGGTTGTTGCTGGTAAATGTGCGTGGGCCAACGTCATTTGAGTCACTACGAAC
TGTTAATGGTGTAATATTCCCAACATATCGTGCTGCATGTGAAGAATTGAAGTTATTAGAAAACGATAGTCATTGGGATACGACAAT
CGCTGAAGCCATTATCTGTGCA

B

MQNRRANNRGQQIDNLRRRTRYLSSADLNRAAFQYDCSNDYSLHPSVCIGQMDVVCEYCGALKFSGETPGLCCLNGKVKLPLLTPPP
EPLYSLLCGETQESRHFLANTQKYNGCFQMTSFGADIIEERGFNPTFKIQGQIHHRIGSLLPFEDTQNKFLQIYFMGNMEEQLDRRL
EINAGMKRAILQDLQCLLHEHHALVRLFKSALERMPSDDYKVVIKADKRPSGTHERTFNAPTVDEVAILIVGEQLEKRDIVLTRRDT
GQLQQISETHRSYDTLQYPLMFWQGEDGYYFNIKMKNPLNGEETTKKVSSMNYYAYRLMIRQNADNYLLRFRRLFQQYCVDMYVKIE
TERLTFIRLNQAKLRSEEYIHLRDAVNTEGNAANIGRLTILPATYIGSPRHMHEYAQDAMTYVRHYGRPDLFITFTCNPKWIEITQL
LLPGQTSSDRHDITARIFRQKIRSLMNFIVKQRVFGDTRCWMYSIEWQKRGLPHAHILIWLVERIQPDQIDDIICAEIPDYEVDPDL
HDVVTTNMIHGPCGAINPQSPCMVDGKCSKRYPRKLTAETVTGNDGYPLYRRRSPDDNGRTVTTKVKRMDFVVDNSWIVPYSPLISK
SFKTHCNVEYCNSVKSIKYICKYVTKGSDMAVFGLQSSNTNDEISRYQVGRYVNCNEAIWRIFAFPIHERHPTVTHLAVHLENGQRV
YFTASNATQRAETPPATTLTSFFAICQSDQFARTLLYSEMPRYYTWNASSKNFQRRKQGDAVPGYPDVRSTDALGRMYTVHPKNNEC
FYLRLLLVNVRGPTSFESLRTVNGVIFPTYRAACEELKLLENDSHWDTTIAEAIICASPSQIRTLFAIIISTCFPSNPCNLWHKYKD
NMSEDILHQIRVSSRNHDIEMNEEIHNRALLLIEDMCYLMCGNLLIRLGMPAPYREMNDAFNRELEREREYDHQELDLVVQTNVPLL
NYQQKKVYDTLMKAIDDENGGLYFLDAPGGTGKTFLMSLVLATVRARSNIAVAVASSGIAATLLEGCPMAKVLAVSKIIIWDECTMA
HKRALEALNRTLKDLRNDSRCFGGAMILLSGDFRQILPVIPRSTAADEINACLKSSNLWRHVKKLQLTTNMRVALLNDTSAEDFSEQ
LLTIGNGQVPVDESSGLISFPNNFCNFVSSKDELINNVFPEIISNYKNYEWMSERAILAAKNKDVDDLNNIIQNKIIGTMHSFKSID
CVTNEDEATNYPIEFLNSLDVPGLPPHNLRLKVGSVVIMLRNINQPKLCNGTRLVVRKLMNNVIYATIMIGKFKGEQVLIPRIPMIP
TDMPFEFKRLQFPIRLAFAMTINKSQGQSLKVCGLNLEHSCFSHGQLYVACSRVGRPSALFVFAPDNKTKNVVYHKVLK
